# Lysosomal permeabilization by Group A *Streptococcus* releases proteins into the macrophage cytosol

**DOI:** 10.1128/iai.00199-26

**Published:** 2026-04-30

**Authors:** Ava Quezada, Kevin Lord, Cheldon Alcantara, Claire Delahunty, Kevin Kim, Olivia Okamoto, John R. Yates, Cheryl Y. M. Okumura

**Affiliations:** 1 Biology Department, Occidental College 5121, Los Angeles, California, USA; 2 Department of Integrative Structural and Computational Biology, Scripps Research Institute, La Jolla, California, USA; University of Illinois Chicago, Chicago, USA

**Keywords:** Group A *Streptococcus*, lysosome, macrophage, streptolysin O (SLO), IL-1β, histones, inflammasome

## Abstract

The human-specific bacterial pathogen Group A *Streptococcus* (GAS) is a significant cause of morbidity and mortality due to its ability to cause severe invasive infection. Although macrophages are important for controlling GAS infection, we and others have demonstrated that GAS can persist in macrophages by perforating the phagolysosome using the pore-forming toxin streptolysin O (SLO). In this study, we examined how phagosomal perforation releases lysosomal and bacterial proteins into the cytosol and alters cytosolic protein content in the macrophage. Using IL-1β as a measure of intracellular pathogen detection, we confirmed that cytosolic preparations from macrophages infected with either wild-type (WT) or SLO-deficient (ΔSLO) bacteria contained new proteins that are absent in uninfected cytosol controls. Proteomic analysis revealed distinct cytosolic protein profiles in both WT- and ΔSLO-infected macrophages. M1 protein was detected only in the cytosol of WT-infected macrophages and corresponded with the IL-1β response, indicating SLO-mediated release of M1 protein from the phagosome, and providing a mechanism for cytosolic recognition of this virulence factor. Unexpectedly, cytosolic extracts of both WT- and ΔSLO-infected macrophages contained histone proteins H1–H4, suggesting that nucleosomal complexes are released into the cytosol during GAS infection. Our work reveals both a mechanism for the activation of the inflammatory response on a cellular level, and the surprising consequences of phagosomal perforation in GAS infections. These responses may collectively contribute to the pathologies observed during severe invasive GAS infection, and can help inform therapies aimed at improving macrophage function and patient outcomes.

## INTRODUCTION

In addition to millions of annual cases of non-invasive infections, Group A *Streptococcus* (GAS; *Streptococcus pyogenes*) can cause severe invasive GAS (iGAS) infections, such as cellulitis, bacteremia, pneumonia, streptococcal toxic shock syndrome, and necrotizing fasciitis. In 2023, as part of a post-pandemic increase in iGAS infection, there were an estimated 43,100 life-threatening infections in the United States (1, 2). A similar rise in iGAS infection was seen in Europe, concurrent with the rise and spread of M1_UK_ GAS (3–6). Severe inflammation is associated with iGAS, particularly in necrotizing fasciitis (7), and increased inflammation markers in plasma have been observed in pediatric iGAS cases (8). Although GAS continues to be susceptible to beta-lactam antibiotics, efficient identification and treatment of iGAS, where disease can progress quickly, remains a challenge (9).

Macrophages are key first responder immune cells essential for limiting the spread of GAS infection from soft tissues (10–12). In a human challenge trial of pharyngitis, monocytes, the precursor cell of macrophages, were the dominant responsive cell type and mediated a strong pro-inflammatory response (13). In their role as professional phagocytes, macrophages quickly engulf pathogens in phagosomes, and lysosomal fusion delivers proteolytic enzymes to facilitate bacterial destruction. We and others have shown that GAS, a human-specific pathogen that co-evolved with our immune system, has multiple mechanisms for surviving this macrophage response. These include (i) perforating the lysosomal membrane using the pore-forming toxin Streptolysin O (SLO) or recruiting the host protein CD63/LAMP-3, which releases ions and lysosomal proteins into the cytosol, and (ii) inhibiting v-ATPase activity, preventing lysosomal acidification (14–16). Following lysosomal permeabilization, GAS remains in lysosomes, where it neither replicates nor escapes into the cytosol, enabling persistence in a cell that normally functions to kill them (7, 11, 16, 17).

While GAS-mediated perforation of the phagolysosomal membrane allows bacterial survival, it also permits the release of lysosomal contents into the macrophage cytosol. In our previous work, we demonstrated that SLO causes the leakage of proteins up to 40 kD in size into the cytosol, including active cathepsin B, a lysosomal enzyme (16). The presence of cathepsin B in the cytosol acts as a danger signal that can trigger a secondary host response, such as activation of the NLRP3 inflammasome, resulting in the proteolytic processing and secretion of IL-1β (18, 19). This secondary response in macrophages may serve as an important safety net mechanism to facilitate the clearance of non-functional macrophages and recruit additional immune cells for pathogen clearance. It is well-established that GAS induces NLRP3 inflammasome activation, and this activation is proposed to be protective in invasive infections (20, 21). In previous reports, the GAS M1 surface protein has been shown to stimulate NLRP3 activation (22). Additionally, the cysteine protease SpeB can directly cleave pro-IL-1β in an NLRP3-independent manner to generate an inflammatory response (21, 23). In both cases, bacterial proteins must be delivered intracellularly to interact with their targets, but the mechanism of this delivery to the cytosol remains unclear. Furthermore, it is unknown whether the concentrations of these GAS proteins in an individual cell are sufficient to trigger this response. SpeB is a secreted protein that does not accumulate to appreciable levels until GAS are in stationary phase (24).

In this study, we isolated cytosol from infected macrophages to identify both host and bacterial proteins released during GAS macrophage infection and phagolysosomal permeabilization. While we used the IL-1β response as a measure of macrophage second-line responses to infection, our data reveal that GAS infection induces unexpected changes in the cytosolic protein profile, introducing or eliminating proteins that may influence cellular response. These results inform our understanding of bacterial persistence and host response, providing a foundation for designing effective treatment strategies for iGAS infections.

## MATERIALS AND METHODS

### Antibodies and chemicals

LPS derived from *Escherichia coli* K12, recombinant SLO, and CA-074 methyl ester (CA074) were purchased from MilliporeSigma. Antibodies to the following proteins were used in this study: LAMP-1 (Cell Signaling Technology, #9091), LAMP-2 (Abcam, ab25631), Cathepsin B (Cell Signaling Technology, D1C7Y, #31718), Histone H3 (Cell Signaling Technology, D1H2, #4499), Lamin A/C (Cell Signaling Technology, 4C11, #4777), and GAPDH (Santa Cruz Biotechnology, 0411 sc-47724).

### Bacterial strains

Wild-type (WT) GAS strain M1T1 5448 (M1 GAS) was originally isolated from a patient with necrotizing fasciitis and toxic shock syndrome (25). Isogenic mutant strains lacking the *slo* (ΔSLO) and *emm1* (ΔM1) genes were previously described (26, 27). Precise allelic replacement of the genes was sequence-verified for each strain. Group B *Streptococcus* (GBS) WT strain serotype III (COH1) was originally isolated from a neonate with early-onset sepsis (28). Bacterial strains were cultivated in Todd-Hewitt broth (THB) at 37°C. For all experiments, bacteria were grown to log phase in the presence of 1:200 pooled normal human serum (Thermo Fisher Scientific) to opsonize bacteria. Heat-killed (HK) bacteria were prepared by incubating a known concentration of bacteria at 95°C for 10 min, followed by a 15-min opsonization in 1:200 pooled normal human serum at room temperature.

### Cell culture

THP-1 cells were purchased from Sigma (cat. 88081201) and cultured in RPMI supplemented with 10% heat-inactivated fetal bovine serum (FBS) (Cytiva), 2 mM L-glutamine, and 100 U/mL penicillin/100 µg/mL streptomycin at 37°C/5% CO_2_. Cells were differentiated to macrophages using 20–40 nM phorbol 12-myristate 13-acetate (PMA) (MilliporeSigma) 48 h prior to experiments.

### Cytosol isolation and western blot

**Fig 1:**
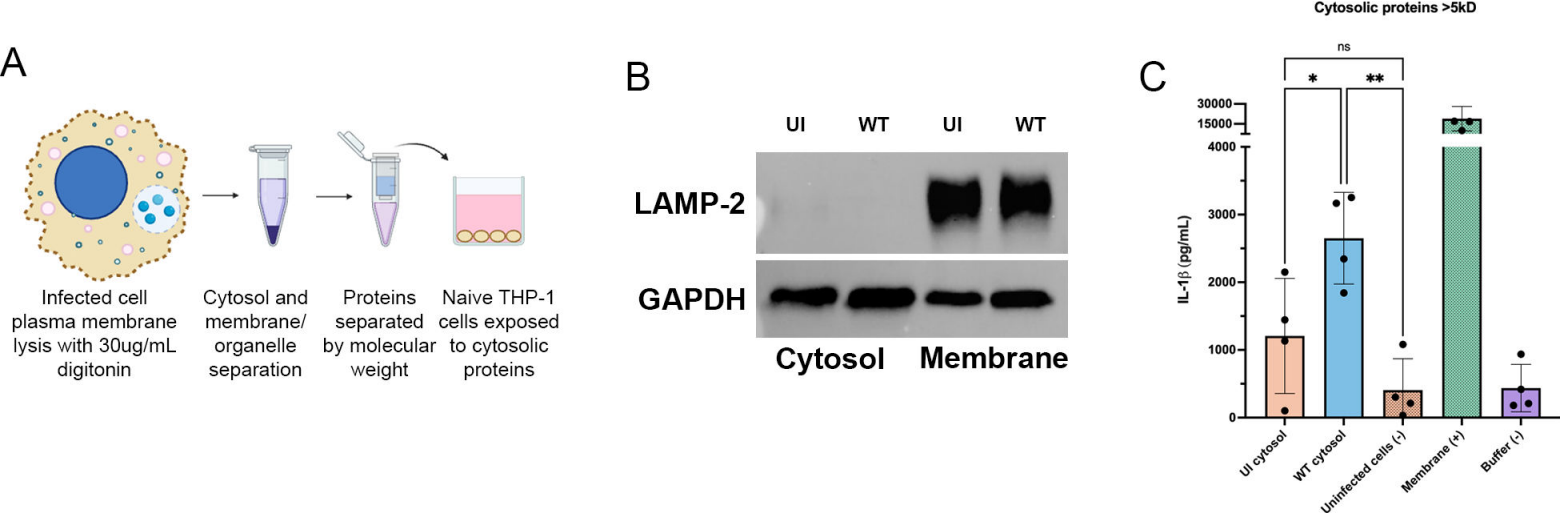
Proteins in GAS-infected cell cytosol stimulate IL-1β secretion. (**A**) Cytosolic protein isolation schematic. Image was created with Biorender (biorender.com). (**B**) Cytosolic and membrane fractions from WT GAS-infected (WT) and uninfected (UI) cells were probed by Western blot for the presence of LAMP-2 (lysosomal membrane protein) and GAPDH (cytosolic protein). (**C**) Cytosolic fractions were tested for inflammasome-activating capacity by exposing naïve THP-1 cells to cytosolic fractions from uninfected (UI cytosol) or WT GAS-infected (WT cytosol) cells and measuring IL-1β secretion by ELISA. THP-1 cells incubated in media only (uninfected cells), with membrane fractions or fractionation buffer included as controls as indicated. Experiments were performed with at least three different cytosolic protein preparations and ELISAs were performed in duplicate for each sample. Data are represented as mean ± SD and analyzed by one-way ANOVA with Tukey’s multiple comparison (*P < 0.05, **P < 0.01, ns = not significant).

Log-phase bacteria were prepared in RPMI supplemented with 2% FBS (infection medium) and added to 10^7^ macrophages at a multiplicity of infection (MOI) of 10. Plates were centrifuged at 480 × *g* for 5 min to initiate and synchronize bacterial contact with cells. Cells were co-cultured with bacteria for 1 h at 37°C, 5% CO_2_. Both culture supernatants (containing detached cells) and attached cells were collected and washed twice with ice-cold PBS. To selectively lyse the plasma membrane (29), cells were incubated with 30 µg/mL digitonin in acetate buffer (50 mM Na-acetate pH 5.6, 150 mM NaCl, 0.5 mM EDTA) supplemented with protease inhibitors (HALT protease inhibitor cocktail, ThermoFisher, 100 µM PMSF, 1 µg/mL pepstatin) and 0.1 µg/mL penicillin for 1 h on ice. Cell lysates were vortexed at maximum speed for 30 s and centrifuged at maximum speed (18,400 × *g*) for 15 min at 4°C to pellet membrane proteins. The pellet consisting of intact organelles including lysosomes was resuspended in acetate buffer and collected as the membrane fraction. The supernatant (cytosol) was filtered through a 0.22-µm filter syringe to exclude contaminating bacteria. Cytosolic fractions were centrifuged in 5 kDa molecular weight cut-off (MWCO) columns at 12,000 × *g* to concentrate samples (Fig. 1C) or 30 kDa MWCO columns at 15,000 × *g* for 10 min at 4°C to separate the content by size (all other data). All fractions were stored at −80°C. Protein concentrations were measured by BCA assay (Thermo Fisher Scientific), and 10 µg of each sample was run on SDS-PAGE gels and transferred to a 0.2-µm PVDF membrane. Blots were blocked with 5% (w/v) non-fat dry milk and probed with the indicated antibodies overnight at 4°C.

### Bacterial phagocytosis and intracellular survival assays

A total of 5 × 10^5^ THP-1 cells were infected with the indicated bacteria at an MOI of 10 in infection medium for an initial period of 30 min. After initial infection, 100 µg/mL penicillin and 50 µg/mL gentamicin were added to the media for 1 h to kill extracellular bacteria. For phagocytosis assays, cells were washed four times with PBS, lysed with 0.025% Triton, and enumerated on Todd Hewitt agar plates. The percent of internalized bacteria was calculated as the number of bacteria remaining in cells after the 1.5-h incubation compared with the initial inoculum. For intracellular infection/survival assays, cells were incubated for additional time as indicated in the presence of antibiotics. Intracellular inoculum was calculated as the number of bacteria remaining in cells after the initial 1.5-h incubation period, and survival of intracellular bacteria was calculated as the number of bacteria remaining at the indicated time point compared with the intracellular inoculum.

### Immunofluorescence

A total of 2 × 10^5^ THP-1 cells grown on coverslips were infected with the indicated bacteria at an MOI of 10 in infection medium for 1 h. Cells were fixed with 4% paraformaldehyde without permeabilization. Samples were blocked and incubated with additional pooled normal human serum to re-label bacteria, followed by incubation with goat anti-human IgG Alexa Fluor 488 antibody (ThermoFisher Scientific). Samples were permeabilized with 0.1% Triton in block solution and incubated with additional human serum to re-label bacteria, followed by incubation with goat anti-human IgG Alexa Fluor 647 antibody (ThermoFisher Scientific). Samples were mounted on slides with ProLong antifade reagent with DAPI (ThermoFisher Scientific). Images were acquired using a 63× objective on a Stellaris 8 confocal microscope (Leica) with 405, 488, and 561 lasers and LAS X software (version 5.3.1).

### ELISA

A total of 5 × 10^5^ THP-1 cells were infected with the indicated live bacteria at an MOI of 10 (live bacterial infection) or the indicated amount of recombinant SLO in infection medium for 2 h. For infection experiments with cytosolic fractions, cytosol fractions were adjusted in cell culture media (RPMI + 10% FBS) to 20 μg per 5 × 10^5^ THP-1 cells and exposed to cells for 24 h. For intracellular infection experiments, cells were prepared as described above, and the culture supernatant was collected from cells to assess IL-1β levels before using the remaining cells to enumerate surviving bacteria. For all experiments, culture supernatants were collected and centrifuged to pellet detached cells and cell debris. Release of human IL-1β into the culture supernatant was determined by enzyme-linked immunosorbent assay (ELISA) (R&D Systems) in accordance with the manufacturer’s instructions. Plates were read at 450 nm with a plate reader. IL-1β was calculated in each treatment in reference to the provided standard. All samples were measured in duplicate, and experiments were repeated at least three independent times.

### Label-free proteomics analysis

Samples were MeOH/CHCl_3_ precipitated, and the pellet was air-dried. Pellets were resuspended in 60 μL of buffer (8M urea, 100 mM Tris, pH 8.5) and reduced with 3 μL of 100 mM tris(2-carboxyethyl) phosphine hydrochloride (TCEP). Samples were alkylated in the dark for 20 min with 250 mM iodoacetamide and digested with trypsin overnight at 37°C. Samples were acidified with formic acid (5%), and 1 μg of sample was loaded onto EvoTips (Evosep) according to the manufacturer’s protocol. Samples were run on an Evosep One (Evosep) coupled to a timsTOF Pro mass spectrometer (Bruker Daltonics). Peptides were separated with a gradient of buffer A (0.1% formic acid in H_2_O) and buffer B (0.1% formic acid in acetonitrile) on a 15 cm × 150 µm ID column with BEH 1.7 μm C18 beads (Waters) and an integrated tip pulled in-house. MS scans were acquired in PASEF mode, with one MS1 TIMS-MS survey scan and 10 PASEF MS/MS scans per 1.1 s acquisition cycle. Both ion accumulation time and ramp time in the dual TIMS analyzer were set to 100 ms, and the ion mobility range was 1/K0 = 0.6 to 1.6 V s cm^−2^. The m/z range was 100–1,700. Precursor ions selected for MS/MS analysis were isolated with a 2 Th window for m/z < 700 and a 3 Th window for m/z >700. Collisional energy was lowered linearly from 59 eV at 1/K0 = 1.6 V s cm^−2^ to 20 eV at 1/K0 = 0.6 V s cm^−2^ as a function of increasing mobility. Precursors for MS/MS were picked at an intensity threshold of 2,500, a target value of 20,000, and an active exclusion of 24 s. Singly charged precursor ions were excluded with a polygon filter. Tandem mass spectra were extracted from raw files using RawExtract (Version 1.9.9) and were searched against a concatenated Human/*Streptococcus pyogenes* database combined_UniProt_NCBI_Human_Streptococcus_pyogenes_03-01-2023 with reversed sequences using ProLuCID. A static modification of carbamidomethylation on cysteine (57.02146) was considered. Data were searched with 50 ppm precursor ion tolerance and 600 ppm fragment ion tolerance. Data were filtered using DTASelect2 to a protein false-positive rate of <1%. A minimum of two peptides per protein and one tryptic end per peptide were required. Statistical models for peptide mass modification (modstat) and tryptic status (trypstat) were applied.

### Proteomic data analysis

Independent preparations of uninfected (UI, four replicates), WT-infected (WT, four replicates), and ΔSLO-infected (SLO, three replicates) cytosolic fractions were analyzed. Contaminants, including keratin and reverse hits, were removed from the data sets before analysis. The data sets were filtered to only include proteins that were identified in at least two of the biological replicates for each sample to yield a list of 1,532 proteins that were confidently identified (Fig. S4, Table S1). Normalized spectral abundance factors (NSAFs) were log2 transformed and normalized by scaling each value against the average of all the proteins within the sample, followed by normalization by correlation slope (30). missForest was used to impute missing values in replicate samples (31, 32), and values below the least expressed protein were imputed for proteins consistently absent in a sample type. Principal component analysis (PCA) was performed using prcomp in R on individual replicates using squared cosine values. Heat maps were generated using heatmaply (33). Proteins of interest were analyzed using ShinyGO 0.82 (34).

### DNA quantification

Total DNA was quantified via a Qubit 3.0 fluorometer using the dsDNA HS Assay Kit (ThermoFisher Scientific). Two microliters of cytosolic fraction or digitonin acetate buffer (control) was diluted in Qubit dsDNA HS reagent/buffer solution. DNA was measured against the provided standard and calculated relative to the amount of protein in the sample. Digitonin acetate buffer spiked with THP-1 genomic DNA was also included as a control (data not shown). All samples were measured in duplicate, and experiments were repeated at least three independent times.

### Statistics

All graph data were analyzed using Prism v. 10.6.0 (GraphPad Software) by one-way ANOVA with Tukey’s multiple comparison tests. Outliers were assessed by the ROUT method (Q = 1%). For all data presented, ****P < 0.0001, ***P < 0.001, **P < 0.01, *P < 0.05, n.s., not significant.

## RESULTS

### Cytosolic fractions from WT GAS-infected cells have inflammasome-stimulating activity

In our previous work, we demonstrated that GAS infection resulted in lysosomal permeabilization and leakage of large lysosomal proteins into the cytosol, including cathepsin B, which can cause activation of the NLRP3 inflammasome (16, 19). To determine which lysosomal and bacterial proteins were released into the cytosol, as well as to determine global cytosolic protein changes in response to infection and lysosomal leakage, we developed a cytosolic protein isolation protocol for proteomic analysis (Fig. 1A). Cells were infected for 1 h with the indicated bacteria to allow lysosomal permeabilization and release of lysosomal contents into the cytosol. Cells were lysed with 30 μg/mL digitonin, which lyses the plasma membrane but keeps organellar membranes intact (29). Cell lysates were spun to separate cytosolic proteins from membrane proteins and intact organelles, then treated with antibiotics and filtered with a 0.2-μm filter to eliminate the presence of live bacteria in the sample (data not shown). Samples were concentrated in columns with a 5 kD molecular weight cut-off and analyzed by Western blot (Fig. 1B). The absence of the lysosomal membrane protein LAMP-2 confirmed the resulting cytosolic fractions were free of intact lysosomes (Fig. 1B). GAPDH was found in high abundance in the cytosolic fraction as expected, and was also present in the membrane fraction since not all cytosol was collected to prevent disturbance of the membrane pellet (Fig. 1B). We next tested whether our cytosolic fractions had inflammasome-stimulating activity by exposing naïve PMA-differentiated THP-1 macrophages to cytosolic fractions and measuring IL-1β secretion (Fig. 1A). We hypothesized that macrophages could uptake proteins in the cytosolic fractions, similar to exposure to recombinant M1 protein (22). In previous work, stimulation and differentiation of THP-1 cells using PMA provide a sufficient priming signal to initiate inflammasome assembly and pro-IL-1β transcription (signal 1), allowing inflammasome activation and IL-1β secretion by the addition of GAS M1 protein (signal 2) (22, 35). Other cell types, such as mouse bone marrow-derived macrophages, require pre-stimulation with agonists, such as LPS (22, 35). Stimulation with both PMA and LPS caused increased basal IL-1β secretion in uninfected cells and a uniformly high IL-1β response to all cytosolic fractions regardless of infection status (Fig. S1). This is similar to LPS treatment and inflammasome activation in human PBMCs, where additional agonists have no additive effect (22). We therefore tested untreated (no LPS pre-stimulation) PMA-differentiated THP-1 macrophage responses to cytosolic fractions (Fig. 1C). THP-1 macrophages released an increased amount of IL-1β in response to cytosolic fractions from WT-infected cells compared with cytosolic fractions from uninfected cells, confirming that we were capturing proteins in our cytosolic fraction that had inflammasome-stimulating activity (Fig. 1C). Thus, we were able to successfully isolate cytosolic fractions from GAS-infected cells that contained proteins that were leaked from permeabilized lysosomes and had inflammasome-stimulating activity.

### Cytosolic fractions from ΔSLO-infected cells also have inflammasome-stimulating activity

**Fig 2:**
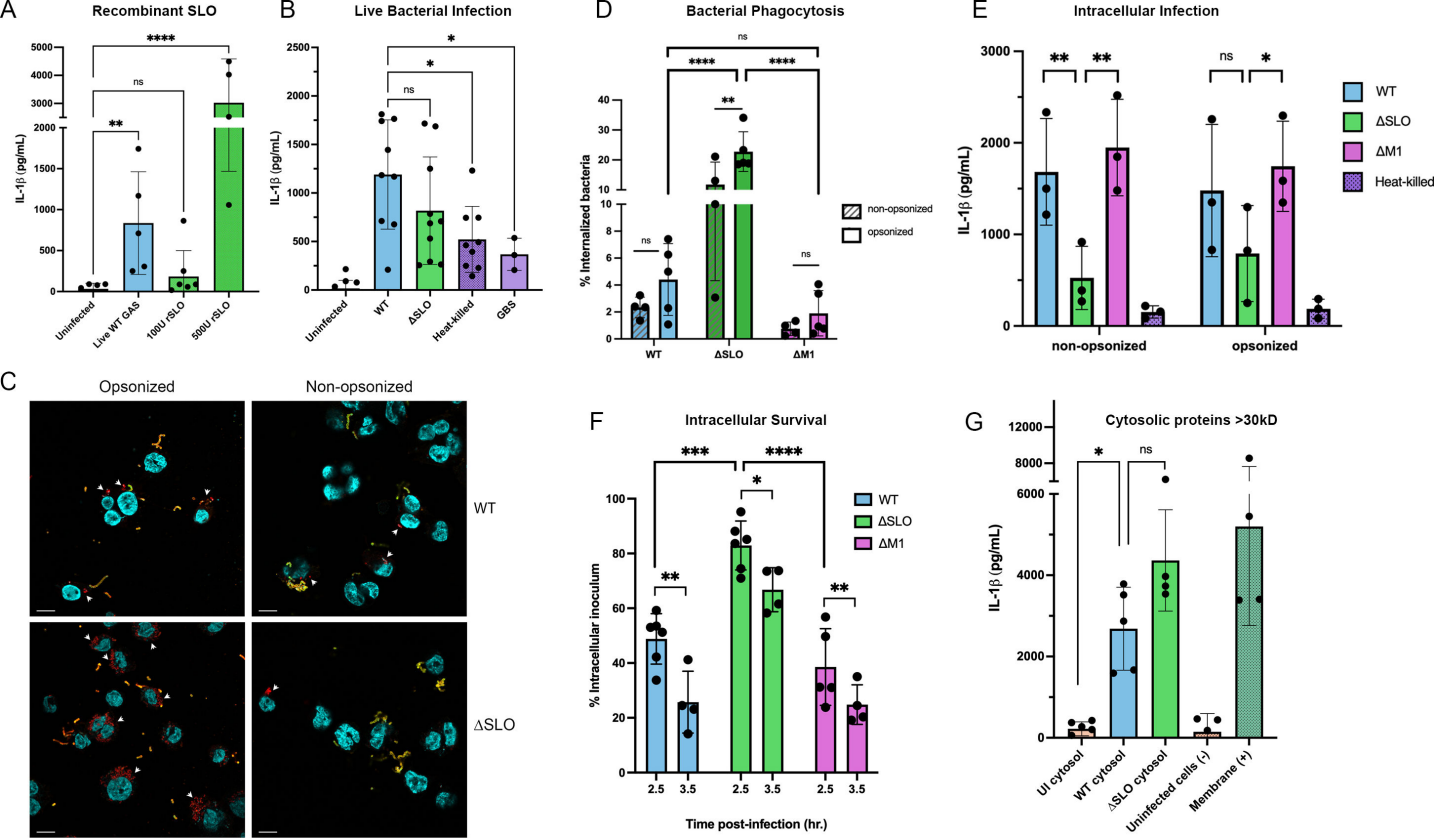
Cytosolic proteins from ∆SLO-infected cells also stimulate IL-1β secretion. IL-1β secretion was measured by ELISA for PMA-differentiated THP-1 macrophages responses to (**A**) recombinant SLO (rSLO) or (**B**) live bacterial infection as indicated. Phagocytosis of non-opsonized and opsonized bacteria was assessed by immunofluorescence (**C**) and by enumeration (**D**). For immunofluorescence experiments, extracellular bacteria were labeled with green and red fluorescence (predominantly green or yellow merge) and intracellular bacteria were labeled with red fluorescence (examples indicated by arrowheads); scale bar = 10 um. Cells exposed to only intracellular bacteria were assessed for IL-1β secretion by ELISA (**E**) or bacterial survival (**F**). (**G**) Cytosolic fractions were tested for inflammasome-activating capacity by exposing naïve THP-1 cells to cytosolic fractions from uninfected (UI cytosol), WT GAS-infected (WT cytosol), or ∆SLO mutant-infected (∆SLO cytosol) cells and measuring IL-1β secretion by ELISA. Experiments were performed at least three independent times (**A–F**) or with at least three different cytosolic protein preparations (**G**), and ELISAs were performed in duplicate for each sample. Data are represented as mean ± SD and analyzed by one-way ANOVA with Tukey’s multiple comparison (*P < 0.05, **P < 0.01, ***P < 0.001, ****P < 0.0001, ns = not significant). For panels D and F, there were statistically significant differences (***P < 0.001) between WT and ∆M1 strains and the ∆SLO strain in the non-opsonized and at 3.5 h post-infection, respectively (data not shown).

The pore-forming toxin SLO permeabilizes the phagolysosomal membrane to allow the release of >40 kD proteins into the cytosol, including cathepsin B, which could stimulate inflammasome activity and IL-1β secretion (16, 19). Others have demonstrated that SLO itself can stimulate inflammasome activity (20, 35). We therefore tested whether the inflammasome-stimulating activity we observed from cytosolic fractions of WT GAS-infected cells (Fig. 1C) was due to the presence of SLO. We found that in the absence of bacteria, recombinant SLO alone did not have inflammasome-stimulating activity until added at high concentrations (Fig. 2A).

We next infected THP-1 macrophages with the live ∆SLO mutant strain (Fig. 2B) for 2 h and measured IL-1β secretion. Previous studies have demonstrated that mutant bacteria lacking SLO do not stimulate NLRP3 inflammasomes to the same extent as WT-infected cells (22, 35, 36). In contrast to previous findings, the inflammasome-stimulating activity was not significantly different between cells infected with WT GAS or the ∆SLO mutant in the aggregate (Fig. 2B), though in individual experiments infection with the ∆SLO mutant strain always produced a slightly lower IL-1β secretion response than the WT strain. Infection for different amounts of time produced similar results, with the ∆SLO mutant strain inducing very high levels of IL-1β secretion after 4 h of infection (Fig. S2A). One possibility for this result may be exposure to a higher-than-expected number of bacteria due to bacterial growth in cell culture medium. When we cultured bacteria in infection medium only (RPMI + 2% FBS), we found that bacteria replicated, but there was no difference in growth between WT and ∆SLO mutant strains (Fig. S2B). We could limit bacterial growth using media without serum (RPMI only, Fig. S2B), but limiting bacterial growth did not yield differences in IL-1β secretion in response to the different GAS strains (Fig. S2C).

In our experiments, we opsonize bacteria by growing log phase cultures with pooled normal human serum to maximize bacterial uptake and ensure bacteria are intracellular (Methods). Opsonization has not been performed in previous experiments using the ∆SLO mutant (22, 35, 36). We demonstrated by immunofluorescence microscopy and enumerating the number of intracellular bacteria after infection (bacterial phagocytosis) that THP-1 macrophages uptake substantially more ΔSLO mutant bacteria compared with WT or ΔM1 mutant bacteria, and that uptake of the ∆SLO mutant is significantly increased with opsonization (Fig. 2C and D). We next assessed IL-1β secretion by cells exposed to only intracellular bacteria by adding antibiotics to the culture medium to kill extracellular bacteria after an initial infection period (Fig. 2E). In agreement with our internalization data (Fig. 2C and D) and with the literature (22, 35, 36), we found differences in IL-1β secretion between macrophages infected with non-opsonized WT and ΔSLO mutant bacteria (Fig. 2E). However, when bacteria were opsonized with human serum, these differences were mitigated, likely due to the higher number of ΔSLO mutant bacteria phagocytosed by macrophages (Fig. 2C through E). Intracellular infection with opsonized bacteria for different amounts of time produced similar results (Fig. S2D), and was similar to infection with total live bacteria (Fig. 2B; Fig. S2A and C ). Concomitantly, there was a higher number of surviving intracellular ΔSLO mutant bacteria compared with WT and ΔM1 mutant bacteria, though all bacterial numbers decreased with longer incubation times (Fig. 2F). Together, our results demonstrate that opsonization maximizes bacterial uptake by macrophages, which allows internalized bacteria to be sensed by the NLRP3 inflammasome.

These data highlighted that there was still inflammasome-inducing activity caused by the ΔSLO mutant. To determine whether this activity was caused by live infection and/or secreted proteins, we infected cells with heat-killed WT GAS, which maintains surface proteins but is incapable of secreting bacterial proteins, including SLO and M1. Heat-killed bacteria were efficiently phagocytosed by macrophages both with and without opsonization (Fig. S3). In agreement with our previous results, infection with heat-killed bacteria, which does not cause lysosomal permeabilization, did not produce a notable IL-1β response (Fig. 2B and E) (16, 20). We also infected cells with live Group B *Streptococcus* (GBS) and found a decreased IL-1β response compared with GAS infection, similar to other work (Fig. 2B) (37). Thus, infection with live GAS caused an IL-1β response in THP-1 macrophages, regardless of SLO expression.

In our previous work, we found that cells infected with ΔSLO mutant bacteria also had permeabilized phagolysosomal membranes, mediated by CD63/LAMP-3 (16). We therefore tested cytosol from cells infected with the opsonized ΔSLO mutant strain for inflammasome-stimulating activity (Fig. 2G). For these experiments and for downstream proteomic analyses, to limit our analysis to larger proteins that are still capable of escaping the lysosome (16), we concentrated cytosolic fractions in 30kD molecular weight cut-off columns. WT-infected cytosol retained inflammasome-activating capacity to the same extent of cytosolic fractions obtained using 5-kD molecular weight cut-off columns (Fig. 1C and 2G). In agreement with our live bacterial infection (Fig. 2B and E), cytosol from ΔSLO-infected cells also elicited a strong IL-1β response (Fig. 2G). Interestingly, the IL-1β response to cytosolic fractions was much stronger compared with infection with live bacteria (compare Fig. 2B and G), which may be a function of concentrating NLRP3-stimulating factors and the time of incubation with cytosolic fractions (Methods). Collectively, our data indicate that GAS-induced permeabilization of phagolysosomes causes an IL-1β response, regardless of whether permeabilization is mediated by SLO or by other cellular mechanisms.

### Proteomic analysis of cytosolic fractions reveals proteins released by GAS permeabilization of the phagolysosome

**Fig 3:**
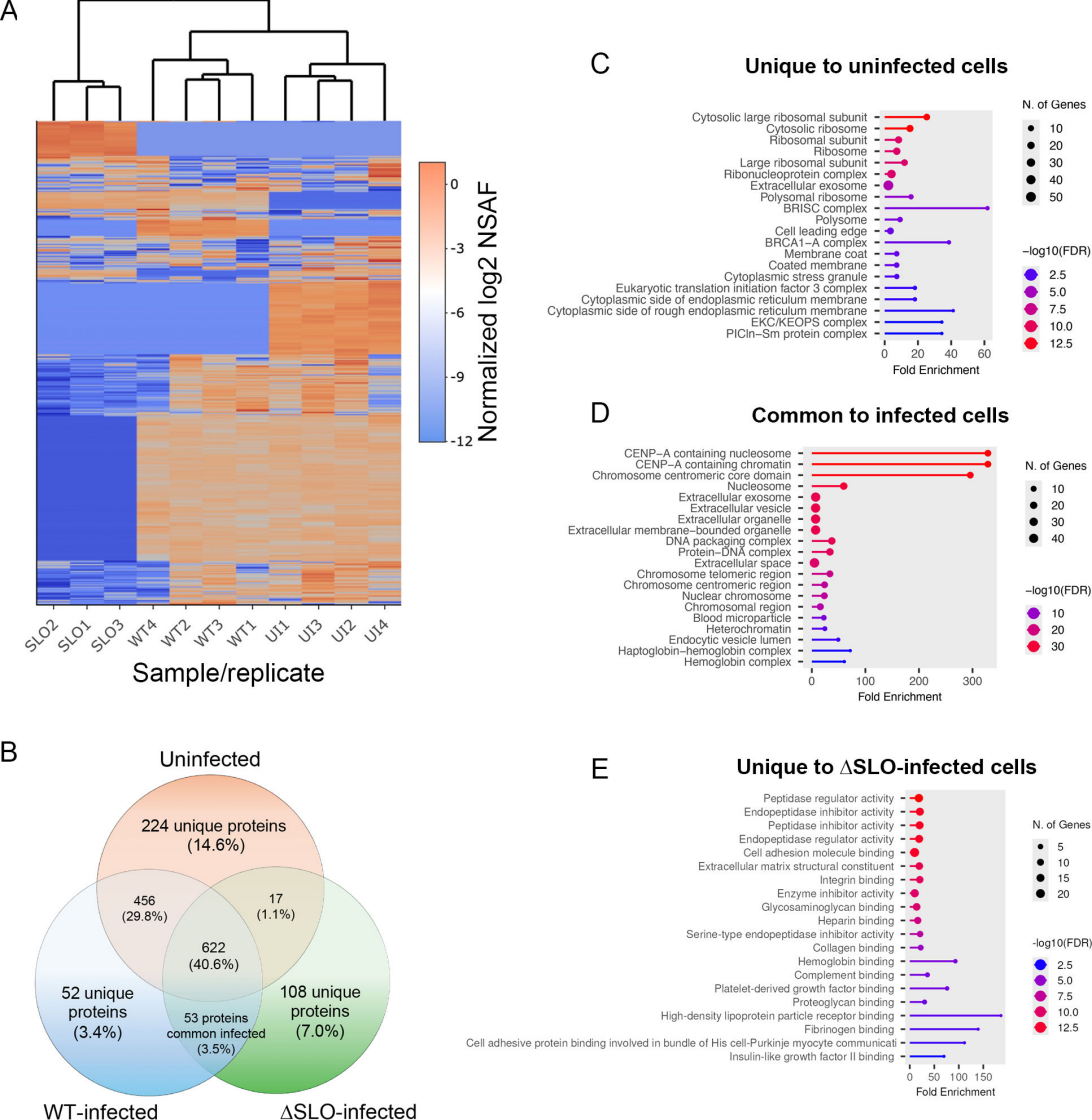
Proteomic analysis of cytosolic proteins from GAS-infected cells. (**A**) Relative abundance of proteins in replicate preparations of cytosol from uninfected (UI), WT- or ∆SLO-infected (SLO) cells. Scale is indicated on the right (blue = absent or low abundance; red = present, high abundance). (**B**) Number of proteins identified in each sample. (**C–E**) Fold-enrichment analysis of proteins in indicated samples. Fold-enrichment, number of genes identified in each pathway and confidence values are indicated. Images were generated in ShinyGO 0.82.

To analyze which proteins may be escaping GAS-permeabilized phagolysosomes, we next performed label-free proteomics analyses on cytosolic fractions isolated from uninfected, WT-infected, or ΔSLO-infected THP-1 macrophages. Across all samples, we confidently identified a total of 1,532 proteins (Table S1, Fig. S4A through C). PCA (Fig. S4D) and heatmap analyses (Fig. 3A) demonstrated that independent preparations of each sample gave reproducible protein profiles. GAS proteins were only found in infected cells as expected (Tables S1 and S2). Cross-referencing the total protein list with GO terms for nuclear matrix (GO:0016363) resulted in a very small list of hit proteins that were not exclusive to the nucleus (Table S2), confirming that our cytosolic fractions were well-isolated and free of organellar contaminants. Notably, the largest differences between samples were the presence/absence of proteins, though there were some differences in expression levels (Fig. 3A and B). We therefore focused our analysis on proteins that were present/absent in our samples (Fig. 3B).

Of the proteins found exclusively in uninfected cell cytosol, surprisingly about 15% were ribosomal subunits or ribosome-associated proteins (Fig. 3C; Tables S2 and S3). Translation initiation factors, DnaJ proteins (molecular chaperones), and ubiquitin-conjugating enzymes were also present in uninfected, but not GAS-infected cytosolic fractions (Tables S2 and S3), indicating that protein translation and folding may be negatively affected in GAS-infected cells. About 15% of proteins unique to uninfected cells were also associated with extracellular exosomes (Fig. 3C; Tables S2 and S3).

In our previous work, we showed that infection with ΔSLO mutant bacteria also results in phagolysosomal permeabilization, supporting that GAS proteins can be found in the cytosol of ΔSLO-infected cells (Table S2). Common to cytosolic fractions from both WT- and ΔSLO-infected cells, we found secreted GAS proteins, including streptococcal GAPDH (Table S2). Proteins annotated as nicotine adenine dinucleotide glycohydrolase precursor and streptolysin O precursor were found in both WT- and ΔSLO-infected cells (Table S2). This annotation may reflect that the proteomic analysis detected the inhibitor of the NADase (encoded by *ifs*) and/or the NADase protein since the ΔSLO mutant was created with precise allelic replacement and retains expression of the other genes in the *ifs-slo-nga* operon (26). Most surprisingly, we found almost all the histone proteins in the cytosol of GAS-infected cells (Fig. 3D; Tables S2 and S3). In addition to the histones, many proteins were also classified as extracellular exosome or vesicle proteins (Tables S2 and S3). Although some of the histone proteins were cross-listed as extracellular exosome proteins, almost half of the proteins common to both WT- and ΔSLO-infected cells were associated with the extracellular exosome. Combined with the data for proteins found exclusively in uninfected cells, this may indicate that GAS infection significantly alters exosome/extracellular vesicles, either in formation or content.

For proteins exclusively found in the cytosol of WT-infected cells, we found lysosomal lumen proteins, as expected given that we have demonstrated SLO pores can induce leakage of dextrans >40 kD (Tables S2 and S3) (16). These proteins included cathepsin B, which we had previously reported to be present in our cytosolic fractions, and lysosomal glycosidases (16). Interestingly, we consistently found M1 protein in WT-infected but not in ΔSLO-infected cells (Table S2), which is likely due to the different-sized pores created by SLO in WT-infected cells or CD63/LAMP-3 in ΔSLO-infected cells.

For proteins exclusively found in ΔSLO-infected cells, we found extracellular matrix (ECM) or ECM-binding proteins, including several collagen proteins and integrins (Fig. 3E; Tables S2 and S3). Immunoglobulin proteins were also present in high abundance (Table S2), along with a number of proteins associated with the complement pathway, including C3, C4, C5, C7, and C8, and complement factors B and H, though it is unclear whether the presence of these proteins was due to opsonizing the bacteria with human serum prior to infection (Tables S2 and S3). Additional GAS proteins were uniquely found in the ΔSLO-infected cytosolic fractions, including the cysteine protease SpeB (Table S2). Our results indicate that phagosomes in ΔSLO-infected cells can be permeabilized, but likely due to the differences in pore size, release different proteins and induce a distinct cellular response from WT-infected cells. Furthermore, our phagocytosis and intracellular survival data indicate that these differences are not due to the presence of less or lysed ∆SLO mutant bacteria in macrophages (Fig. 2C, D and F).

### WT GAS-infected cells allow release of M1 protein into the cytosol to stimulate an IL-1β response

**Fig 4:**
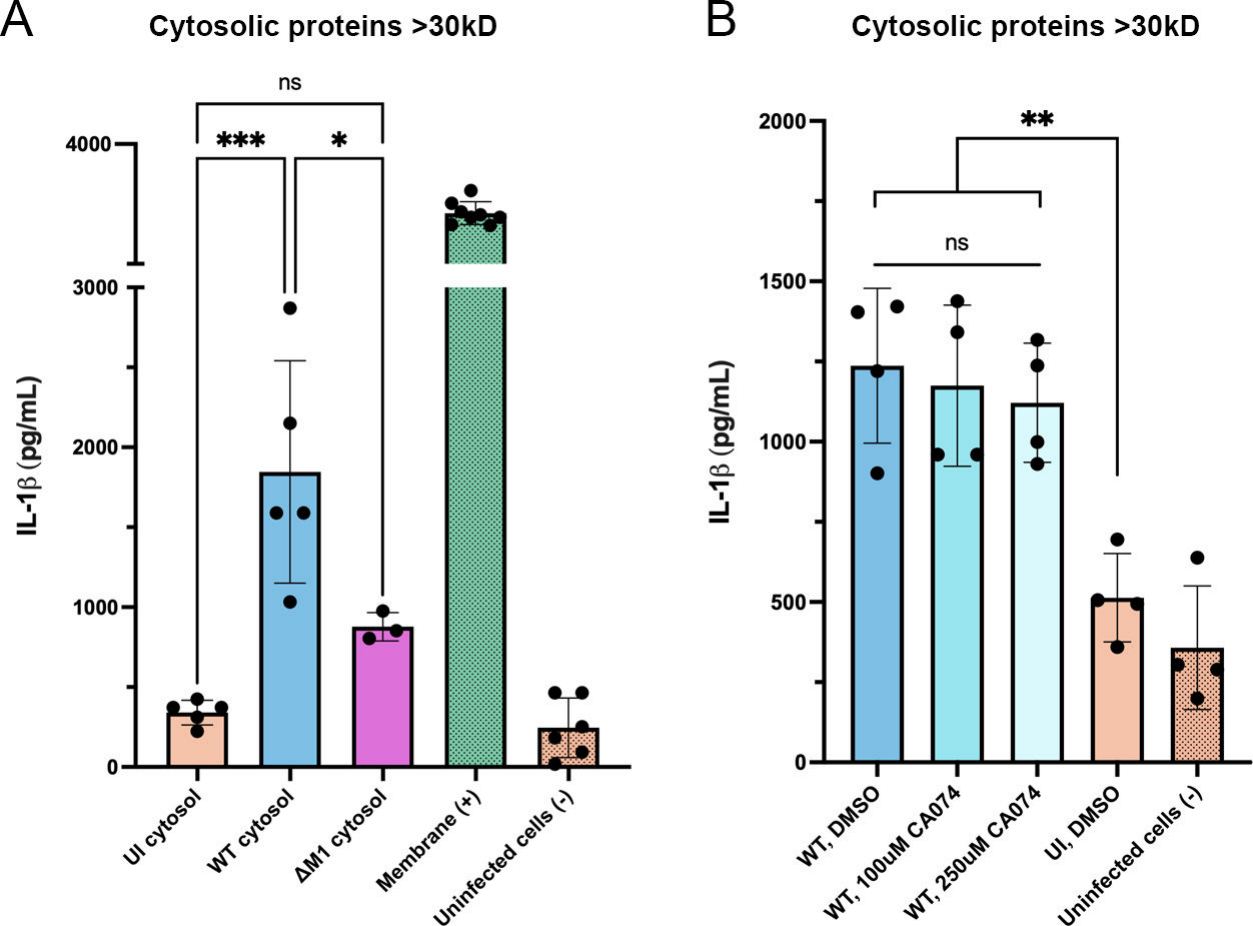
GAS M1 protein, but not lysosomal enzymes trigger an IL-1b response. (**A**) Cytosolic fractions from uninfected (UI), WT-infected, or ∆M1-infected or cells were incubated with naïve THP-1 cells for 2 h. (**B**) Cells were pre-incubated with the indicated concentration of CA-074 Me or DMSO, then exposed to cytosolic proteins from WT-infected or uninfected (UI) cells for 2 h. THP-1 cells incubated in media only (uninfected cells), with membrane fractions or fractionation buffer included as controls as indicated, and IL-1β secretion was measured by ELISA. Experiments were performed with at least three different cytosolic protein preparations, and ELISAs were performed in duplicate for each sample. Data are represented as mean ± SD and analyzed by one-way ANOVA with Tukey’s multiple comparison (*P < 0.05, **P < 0.01, ***P < 0.001, ns = not significant).

In previous work, recombinant M1 protein added to PMA-stimulated THP-1 macrophages could stimulate IL-1β activation and secretion (22). However, it was unclear how M1 could be introduced into the cytosol of macrophages during natural infection. Given that we found M1 protein in the cytosol of WT-infected cells (Table S2), we hypothesized that M1 protein released from WT-permeabilized phagolysosomes could be responsible for the IL-1β secretion we observed (Fig. 1C and 2G). We therefore isolated cytosolic proteins from THP-1 cells infected with the ΔM1 isogenic mutant and tested whether they could stimulate an IL-1β response (Fig. 4A). The ΔM1 mutant is phagocytosed and survives intracellularly to a similar degree as WT GAS (Fig. 2D and F; Fig. S3). In contrast to previous studies (22), we find that the IL-1β response to the ΔM1 mutant is not significantly different compared with the WT strain in both live bacterial infection and intracellular infection experiments (Fig. 2E; Fig. S2C and D). However, THP-1 cells had a significantly decreased IL-1β response to cytosolic preparations from ΔM1-infected cells (Fig. 4A). These results strongly indicate that M1 protein released from WT GAS-permeabilized phagosomes accounts for the majority of the inflammasome activation we observe in response to WT-infected cytosol.

Our previous work showed that cathepsin B released into the cytosol retains activity (16), and others have demonstrated that cathepsin B is capable of stimulating the NLRP3 inflammasome (19, 38). Cathepsin B was not detectable by Western blot in our cytosolic preparations (Fig. S5), which may be due to cathepsin B being close to the molecular weight cut-off we used for concentrating the cytosolic fractions and reducing the amount of detectable cathepsin B in the sample. However, we consistently identified cathepsin B in the cytosol in both WT- and ΔSLO-infected cells by mass spectrometry, which can more sensitively detect low concentrations of protein (Table S2). We therefore tested the ability of our WT-infected cytosolic fractions to induce an IL-1β response in the presence of CA-074 methyl ester (CA-074), a potent and specific cathepsin B inhibitor (38, 39), and found no significant reduction in IL-1β response (Fig. 4B). Combined, our data suggest that M1 protein released from permeabilized phagolysosomes is primarily responsible for the IL-1β response observed in WT-infected cells.

### Histones are released into the macrophage cytosol in response to GAS infection

**Fig 5:**
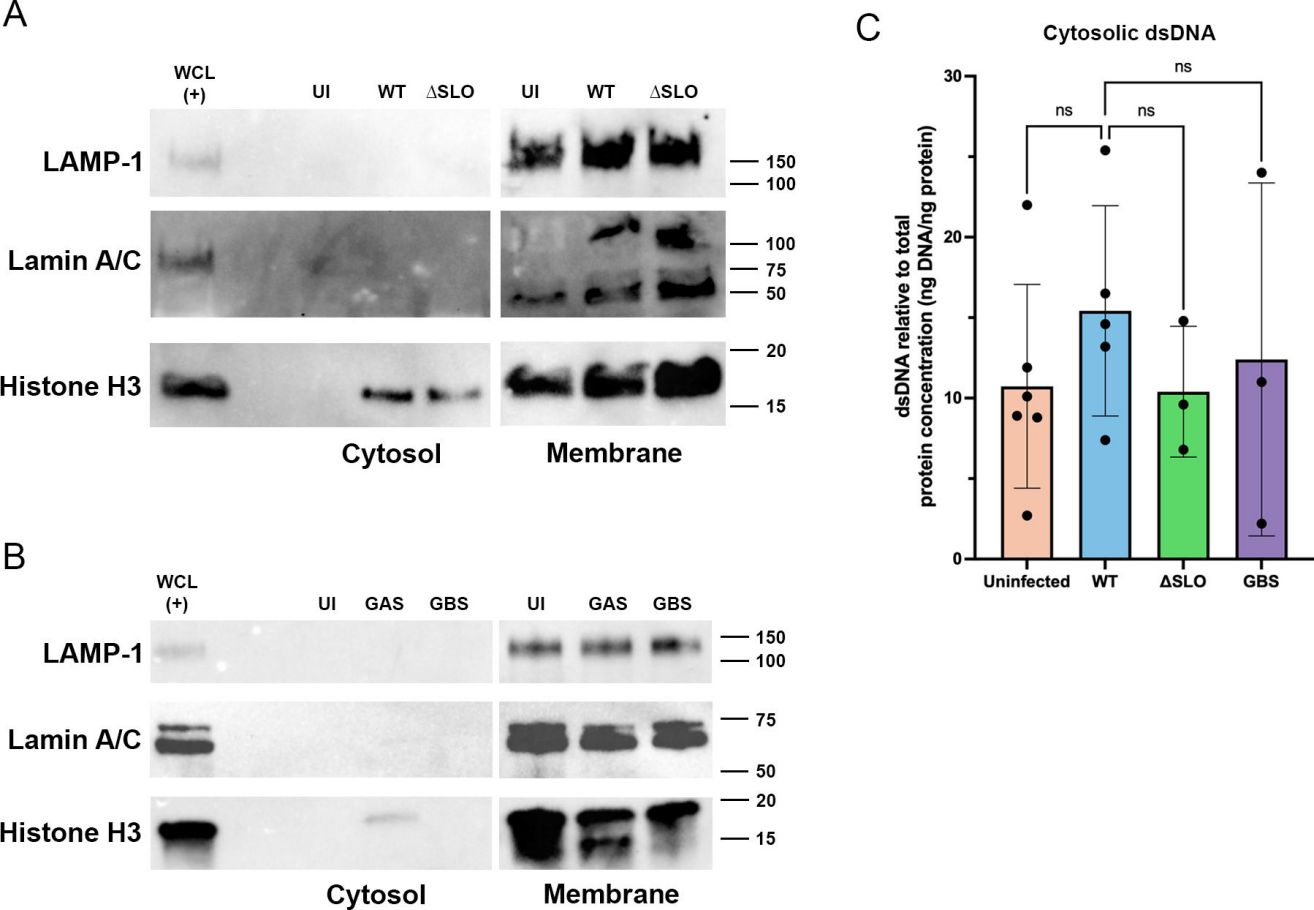
Histones are present in cytosolic preparations of GAS-infected cells. Cytosolic fractions from uninfected (UI), WT GAS-infected, and ∆SLO-infected (SLO) cells (**A**) or GAS-infected (GAS) and GBS-infected (GBS) cells (**B**) were probed with LAMP-1 (lysosome marker, 100–130 kD), Lamin A/C (nuclear marker, 74 and 63 kD respectively), or Histone H3 (17kD) antibodies. Whole cell lysates (WCL) and membrane fractions were included as positive controls, and molecular weights are indicated. Experiments were performed at least three independent times, and representative blots are shown. (**C**) Double-stranded DNA (dsDNA) content in the indicated cytosolic fractions was assessed. DNA amount was normalized to the protein concentration of the sample. Experiments were performed in duplicate at least three independent times, and data are represented as mean ± SD and analyzed by one-way ANOVA with Tukey’s multiple comparison (ns = not significant).

A surprising finding from our proteomic analysis was the identification of histones in the cytosol of both WT- and ΔSLO-infected cells (Fig. 3D; Table S2). The lack of contaminating organellar proteins, including nuclear matrix proteins, in our proteomic analysis suggests that release of these proteins is specific to GAS infection (Table S2). Given the small size of individual histone proteins (10–23 kD), we hypothesize that full nucleosomes are released into GAS-infected cytosol since we used 30-kD molecular weight cut-off columns in the cytosolic preparations and identified almost all histone subunits (Fig. 3D; Tables S2 and S3). To confirm this finding, we performed a Western blot on our cytosolic fractions and looked for the presence of the histone H3 subunit, which was regularly present in WT- but not ΔSLO-infected cytosol (Table S2). Consistent with our findings in our proteomics analysis, we detected histone H3 in the cytosolic fractions of both WT- and ΔSLO-infected cells (Fig. 5A). We did not detect LAMP-1 or Lamin A/C, a nuclear membrane protein, confirming that our cytosolic preparations were not contaminated with organelles, especially nuclei (Fig. 5A). To determine whether histone presence in cytosolic fractions were specifically due to GAS infection, we also prepared cytosol from Group B *Streptococcus* (GBS)-infected cells. We confirmed that histones were only present in the cytosol of GAS-infected cells (Fig. 5B).

The presence of all histone subunits in our infected cytosolic fractions (Table S2) suggests that nuclear DNA could be released from GAS-infected cells. We next quantified DNA in our cytosolic preparations. Although a low amount of DNA was detectable, there was no significant difference in the amount of DNA in cytosolic preparations from uninfected cells compared with GAS- or GBS-infected cells (Fig. 5C). These results are in agreement with previous findings that GAS does not trigger the cytosolic double-stranded DNA sensor cGAS in macrophages (40). Thus, GAS infection causes histones to be present in the cytosol in the absence of DNA, though the mechanism for their presence is unknown.

## DISCUSSION

The human-specific pathogen GAS has co-evolved with the human immune system and has multiple strategies for survival within the host (41, 42). Although typically considered an extracellular bacterial pathogen, GAS can survive in macrophages, altering their functions and immune activity, and using them as shelters against antibiotic treatment (11, 15, 16). Modulating the inflammatory response of macrophages can further promote GAS survival by eliminating microbial competitors and recruiting T cells to the site of infection (41).

In our study, we opsonized bacteria with pooled normal human serum to maximize bacterial uptake and opsonization by macrophages (Fig. 2). Although we did not determine whether there were GAS-specific antibodies in the serum we used, others have demonstrated the presence of immunoglobulins reactive against cell wall components of GAS in intravenous immunoglobulin therapy (IVIG) pooled from the plasma of over a thousand donors (43). Serum also contains complement proteins, which can bind to the surface of GAS and mediate phagocytosis via complement receptors (44). Although GAS has immunoglobulin-degrading enzymes, such as IdeS, which can alter immunoglobulin structure and complement protein binding, such alterations may not interfere with bacterial recognition and phagocytosis by macrophages (44, 45). Notably, opsonization increased the number of intracellular bacteria, especially for the ∆SLO mutant, which subsequently led to a more robust IL-1β response (Fig. 2).

In this work, we determined which proteins have altered expression in the macrophage cytosol during GAS infection, both for regularly expressed cytosolic proteins and proteins that could be introduced through GAS permeabilization of phagosomes such as lysosomal proteins. Although we identified many proteins in our cytosolic fractions as expected, our data sets surprisingly revealed a relatively small number of proteins differentially expressed between infected and non-infected cells (Fig. 3). Furthermore, there is a larger difference in overall protein expression in ΔSLO-infected cells compared with WT-infected and uninfected cells (Fig. 3). These differences in part reflect the size and extent of phagosomal perforation mediated by the different strains of bacteria, as well as the consequences of releasing different proteins into the cytosol (15, 16). The limited number of GAS proteins detected in our proteomics analysis gives confidence in our method that we were not identifying contaminants (Fig. 3; Tables S1 to S3). On the other hand, the proteins we did detect (e.g., M1, GAPDH, SLO, NADase) are secreted proteins that often are studied as vaccine candidates, in part due to the propensity of GAS to produce them (46–48). Other GAS proteins, such as pyruvate dehydrogenase, may be playing a role in modifying the macrophage environment to dampen the inflammatory response (49), but we did not detect those proteins in our cytosolic fractions. Whether such bacterial proteins were below the limit of our detection or play an indirect role in controlling the host response is unknown and should be further studied.

In this study, we used IL-1β response to monitor the release of phagosomal proteins into the cytosol since the NLRP3 inflammasome is a sensor for intracellular danger (22). Although we do not know the exact mechanism for how our cytosolic fractions could induce intracellular detection and inflammasome activation, we hypothesized that THP-1 macrophages could uptake exogenous proteins similar to M1 (22). In antigen-presenting cells, such as macrophages and dendritic cells, proteins can then be exported to the cytosol for cross-presentation (reviewed in reference 50). In previous work, purified recombinant M1 protein could stimulate a strong IL-1β response, but a mechanism for intracellular detection of M1 protein was not determined (22). In contrast to the previous work, we found that infection with live ∆M1 mutant bacteria elicited a robust IL-1β response (Fig. 2) (22). However, given that our ∆M1-infected cytosolic fractions yielded a weak IL-1β response, our data strongly support that M1 protein can be secreted or leaked from perforated WT-infected phagosomes into the cytosol (Fig. 4), providing a mechanism by which M1 can trigger an NLRP3 inflammasome response during natural infection.

In WT infections, release of both M1 and SLO into the cytosol could contribute to the IL-1β response we measured (Fig. 2 and 4; Table S2). While we do not have a double ∆SLO-M1 mutant, our experiments using heat-killed bacteria indicate that while efficiently phagocytosed, cells do not mount a strong IL-1β response to bacteria that cannot actively secrete proteins (Fig. 2B and E; Fig. S3). Surprisingly, however, cytosolic fractions from ΔSLO-infected cells elicited a strong IL-1β response (Fig. 2). In both WT- and ΔSLO-infected cytosol, we found cathepsin B (Table S2), which has been shown to process pro-IL-1β (19). However, using the cathepsin B-specific inhibitor, we showed that this is likely not contributing to the IL-1β response we observe (Fig. 4). In previous work, we observed that ΔSLO bacteria also permeabilize phagosomes using CD63/LAMP-3 (16). This likely produces smaller pores than SLO, preventing larger proteins such as M1 from being released, but allowing other bacterial proteins to escape (16). We did find the GAS cysteine protease SpeB in the cytosol of ΔSLO-infected cells (Table S2), which has been shown to directly process pro-IL-1β (23), and may explain the ability of the ΔSLO mutant to elicit the IL-1β response.

A surprising finding from our data were the presence of histones in the cytosol (Fig. 3 and 5). GAS is a well-known inducer of neutrophil extracellular traps (NETs), where extracellularly released histone-associated DNA mixes with antimicrobial compounds to trap and kill pathogens (51, 52). Release of extracellular histone-associated DNA by macrophages in bacterial infection has been documented (23, 53). Extracellular trap formation requires the breakdown or pore-formation in the nuclear envelope, and such damage could be mediated by SLO, the NLRP3 inflammasome, or gasdermin D, which can be activated by the GAS enzyme SpeB (23, 26, 53, 54). GAS infection does not cause DNA to be present in the cytosol of cells, preventing detection by the double-stranded DNA sensor cGAS (Fig. 5) (40). However, DNA could be degraded by GAS DNase (51), leaving histones in the cytosol. Although we performed a preliminary analysis for nuclear modifications of cytosolic histones such as methylation, we could not determine whether histones in our cytosolic fractions were of nuclear origin due to low concentration (Fig. 5). Thus the origin and mechanism of histone release in GAS infection remains to be determined. Histone proteins have antimicrobial activity against GAS, and inflammatory capacity when released extracellularly, which may be responsible for sepsis during severe invasive infections (52, 55, 56) and thus warrants further study.

Inflammation during GAS infection can have varying consequences depending on the anatomic location. Inflammation in the nasopharynx, for example, is beneficial for both eliciting a strong protective immune response and promoting GAS survival, while inflammation in soft tissues, such as the skin, can result in the severe pathologies associated with iGAS infection (21, 23, 41). Furthermore, persistent IL-1ꞵ activation as a result of GAS infection has been linked with ARF and rheumatic heart disease (57). However, caution must be exercised in using anti-inflammatory therapies, as IL-1 suppressants, such as Anakinra, are associated with increased iGAS outcomes (21). Our work demonstrates that multiple proteins, contributed both by the bacteria and the host, trigger the IL-1ꞵ response as a result of phagosomal permeabilization and the failure of macrophages to degrade and kill GAS. Therapies for GAS infection should therefore be aimed at the restoration of phagosome function, rather than the subsequent inflammatory response, to allow individuals to mount a proper protective immune response.

## Data Availability

Mass spectrometry spectra and analysis parameters are available in the MassIVE database, accession number PXD069795.

## SUPPLEMENTAL MATERIAL

Supplemental material iai.00199-26-s0001.pdf Supplemental methods; Fig. S1 to S5.

Table S1 iai.00199-26-s0002.xlsx Full list of proteins identified in proteomics analysis.

Table S2 iai.00199-26-s0003.xlsx List of proteins in each cytosolic fraction.

Table S3 iai.00199-26-s0004.xlsx Pathway enrichment analyses.

ASM does not own the copyrights to Supplemental Material that may be linked to, or accessed through, an article. The authors have granted ASM a non-exclusive, world-wide license to publish the Supplemental Material files. Please contact the corresponding author directly for reuse.
