# Supplementary material for: Lysosomal permeabilization by Group A Streptococcus releases proteins into the macrophage cytosol
Source: Infect Immun. 2026 Apr 30;94(6):e00199-26. doi: 10.1128/iai.00199-26 (PMC13248697; doi:10.1128/iai.00199-26)

Lysosomal permeabilization by Group A *Streptococcus* releases proteins into the macrophage cytosol

Ava Quezada[a], Kevin Lord[a], Cheldon Alcantara[a], Claire Delahunty[b], Kevin Kim[a], Olivia Okamoto[a], John R Yates III[b], Cheryl YM Okumura[a#]

Supplemental data

Supplemental methods

LPS incubation

THP-1 cells were pre-treated with 10ng/mL lipopolysaccharide from *Escherichia coli* K12 (LPS, MilliporeSigma) for signal 1 enhancement and incubated for 24 hours at 37°C, 5% CO2. Following this incubation, cells were washed with PBS and cytosolic fractions were added to cells. Culture supernatants were assessed for IL-1β as described.

Bacterial growth

Bacteria were resuspended to 5 x 10^6 cfu per well in 24 well plates in the indicated medium. Bacteria were incubated at 37°C/5% CO_2 for the indicated times. At each time point, bacterial cultures were triturated and enumerated on Todd Hewitt agar plates.

Supp. Figure 1: THP-1 cells pre-stimulated with LPS secrete a high basal level of IL-1 β. THP-1 cells were incubated with 10ng/mL *E. coli* LPS (+LPS) or media only (-LPS)

24 hours before incubation with cytosolic fractions from WT GAS-infected (WT) or uninfected (UI) cells. THP-1 cells incubated in media only (uninfected cells), membrane fractions or fractionation buffer were included as controls. Data are represented as mean ± SD and analyzed by one-way ANOVA with Tukey's multiple comparison (*$p<0.05$, **$p<0.01$, ns = not significant). A significant effect of LPS was found by two-way ANOVA (****$p<0.0001$).

Supp. Figure 2: IL-1β response to opsonized bacteria under various conditions. IL-1β secretion was measured by ELISA for PMA-differentiated THP-1 macrophages responses to (**A**) total live bacterial infection for varying times, (**C**) total live bacterial infection in different culture media or (**D**) cells exposed to intracellular bacteria for varying times. (**B**) Bacterial growth in RPMI supplemented with 2% FBS or RPMI only in the absence of cells. For all panels, experiments were performed at least 3 independent times and ELISAs were performed in duplicate for each sample. Data are represented as mean ± SD and analyzed by one-way ANOVA with Tukey's multiple comparison (ns = not significant).

Supp. Figure 3: Uptake of bacteria by THP-1 macrophages. Non-opsonized or opsonized ∆M1 mutant or heat-killed (HK) bacteria were incubated with THP-1 cells for 1 hour. Extracellular bacteria were labeled with green and red fluorescence (predominantly green or yellow merge) and intracellular bacteria were labeled with red fluorescence (examples indicated by arrowheads). Scale bar = 10um.

Supp. Figure 4: Number of proteins identified in each independent preparation of cytosolic fractions of A) uninfected (UI), B) WT-infected (WT) and C) ΔSLO-infected (SLO) cells. Number of proteins common to at least 2 replicates and number of unique (or low abundance) proteins are indicated. Images were generated with Venny v.2.1 (https://bioinfogp.cnb.csic.es/tools/venny/). D) PCA analysis of all individual preparations.

Supp. Figure 5: Cathepsin B is not detectable by Western blot in cytosolic fractions. Cytosolic proteins >30kD were isolated from uninfected (UI) or WT-infected THP-1 cells and probed for LAMP-1 (lysosomal membrane protein, 90-120kD), Cathepsin B (lysosomal lumen protein, 27kD) or GAPDH (cytosolic protein, 37kD). The membrane fraction from WT-infected THP-1 cells was included as a control. Experiments were performed at least 3 independent times, and a representative blot is shown.

Supp. Table 1: All data from the proteomics of cytosolic fractions collected from uninfected (UI), WT-infected (WT) and ΔSLO-infected (SLO) THP-1 macrophages.

Supp. Table 2: Selected proteomics data of unique proteins from cytosolic fractions collected from uninfected (UI), WT-infected (WT) and ΔSLO-infected (SLO) THP-1 macrophages.

Supp. Table 3: Pathway enrichment analyses of selected proteomics data of unique proteins from cytosolic fractions collected from uninfected (UI), WT-infected (WT) and ∆SLO-infected (SLO) THP-1 macrophages.

Supplemental Fig. 1

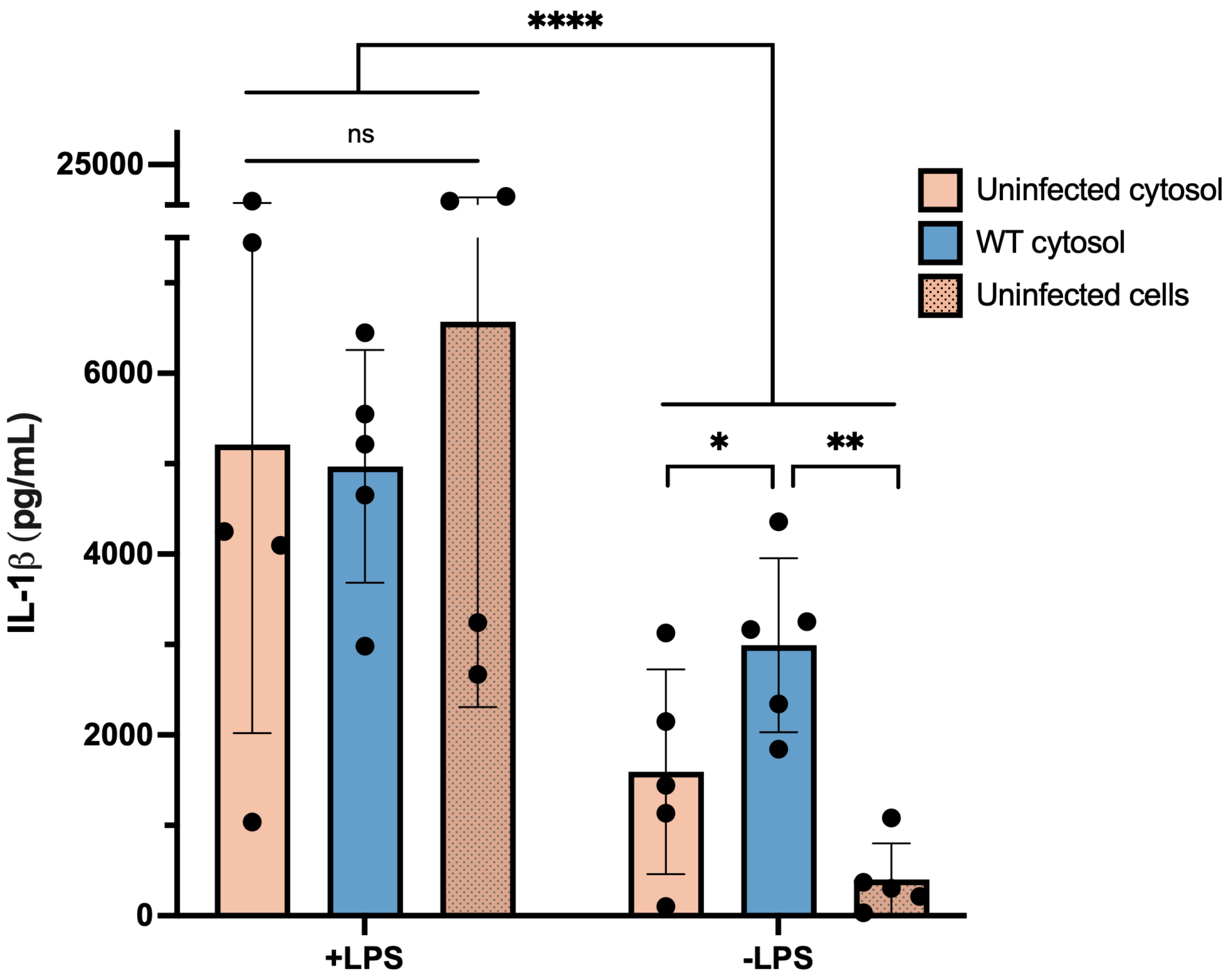

Supplemental Fig. 2

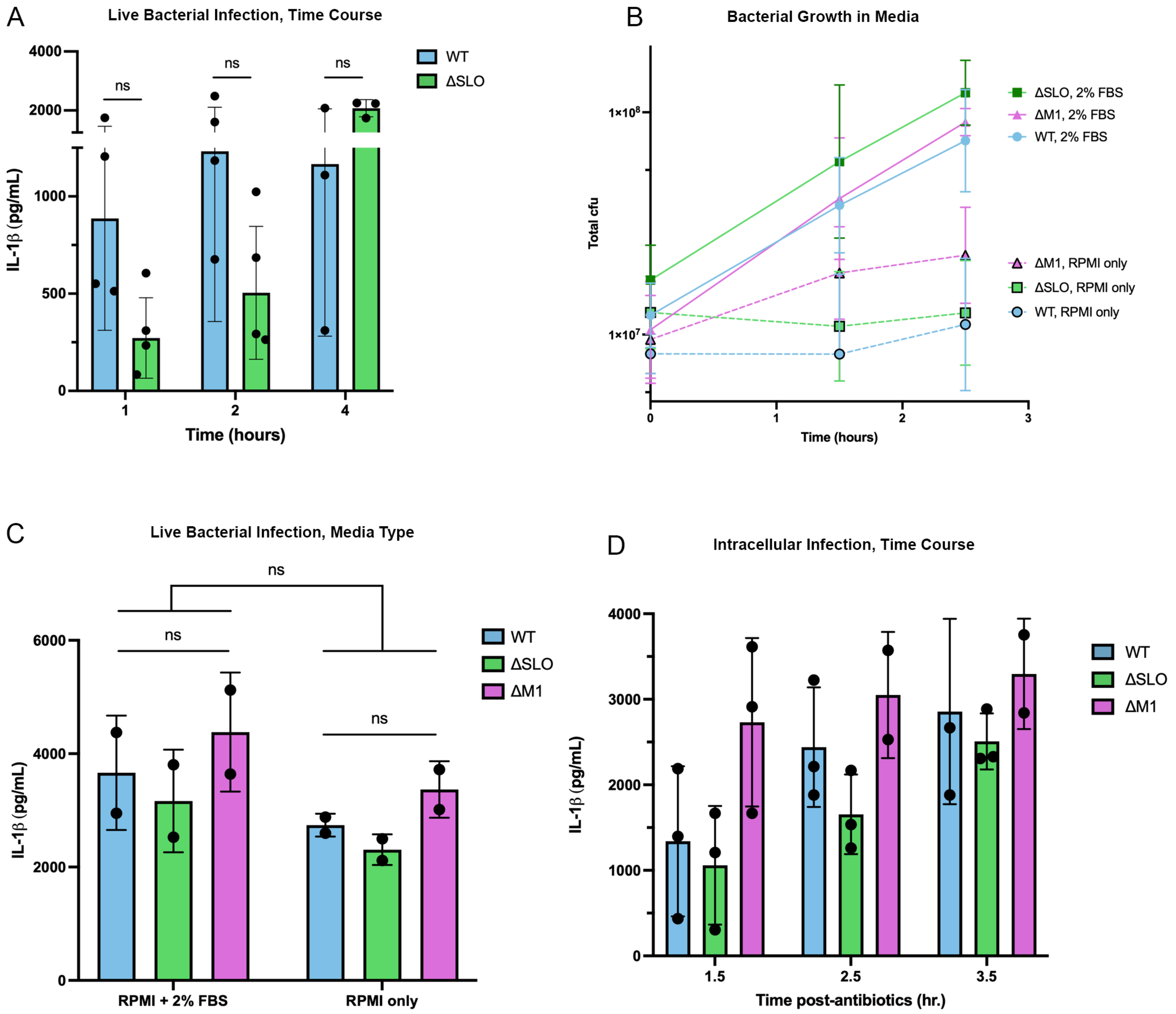

Supplemental Fig. 3

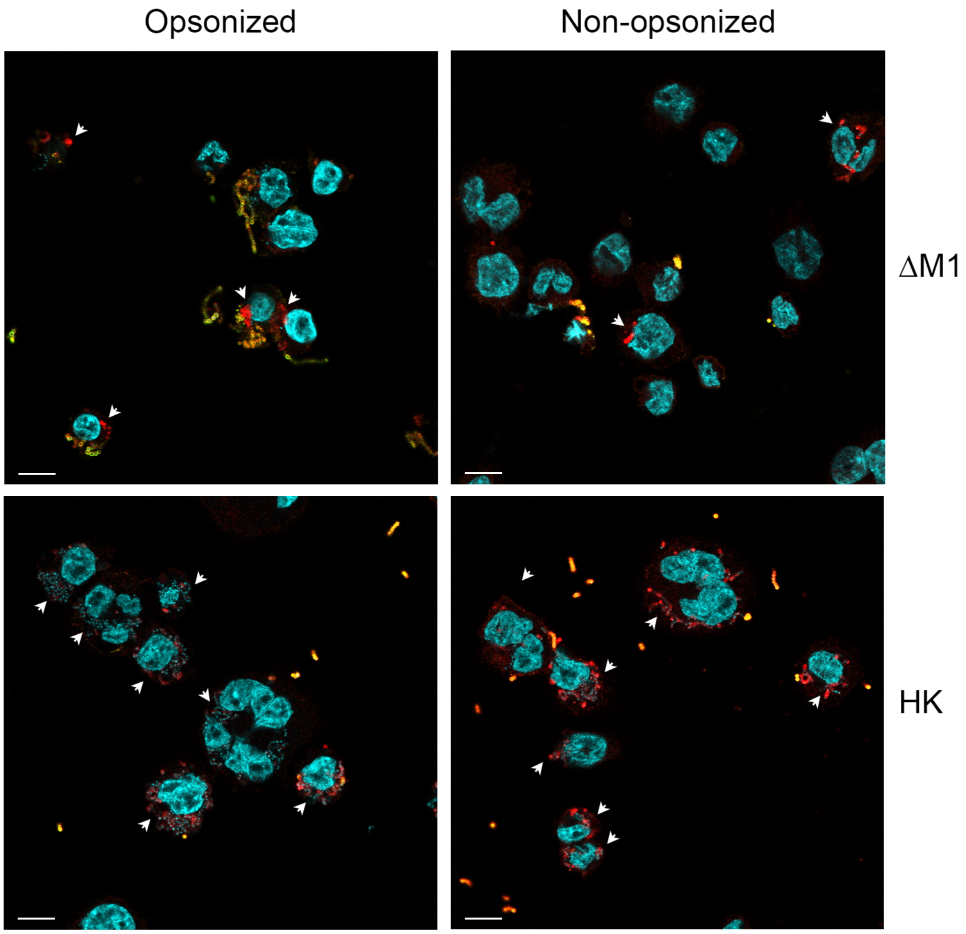

Supplemental Fig. 4

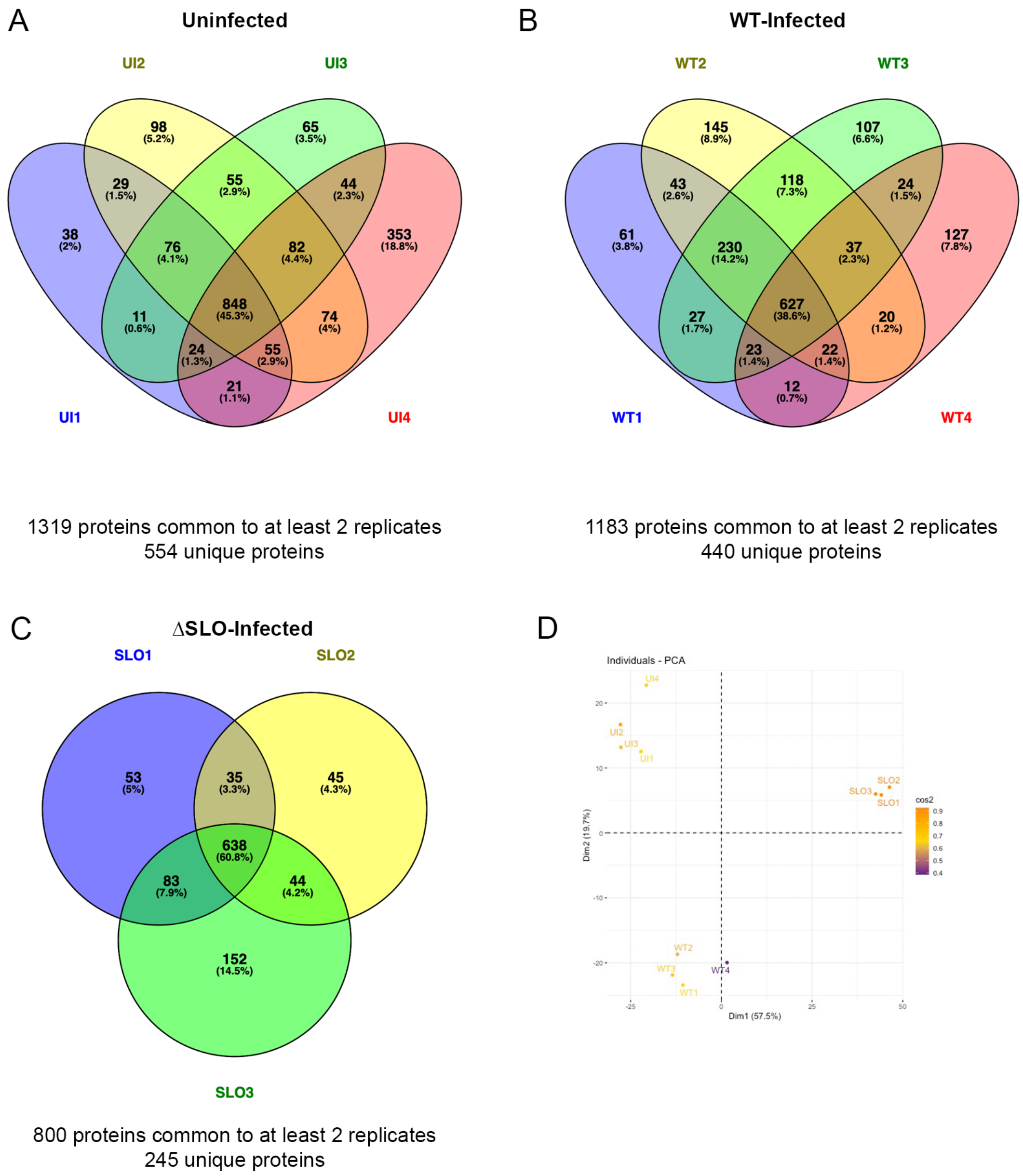

Supplemental Fig. 5

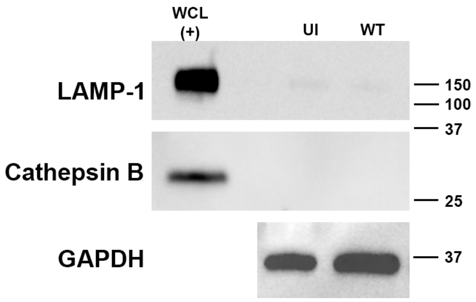

Supplement: Supplemental material — Supplemental methods; Fig. S1 to S5. [file iai.00199-26-s0001.pdf]
